# Simultaneous silencing of isoamylases ISA1, ISA2 and ISA3 by multi-target RNAi in potato tubers leads to decreased starch content and an early sprouting phenotype

**DOI:** 10.1371/journal.pone.0181444

**Published:** 2017-07-14

**Authors:** Stephanus J. Ferreira, Melanie Senning, Michaela Fischer-Stettler, Sebastian Streb, Michelle Ast, H. Ekkehard Neuhaus, Samuel C. Zeeman, Sophia Sonnewald, Uwe Sonnewald

**Affiliations:** 1 Department of Biology, Friedrich-Alexander-University Erlangen-Nuremberg, Erlangen, Germany; 2 Department of Biology, ETH Zürich, Zürich, Switzerland; 3 Department of Biology, Division of Plant Physiology, Technical University Kaiserslautern, Kaiserslautern, Germany; Shanghai Institutes for Biological Sciences, CHINA

## Abstract

Isoamylases hydrolyse (1–6)-alpha-D-glucosidic linkages in starch and are involved in both starch granule formation and starch degradation. In plants, three isoamylase isoforms with distinct functions in starch synthesis (ISA1 and ISA2) and degradation (ISA3) have been described. Here, we created transgenic potato plants with simultaneously decreased expression of all three isoamylases using a chimeric RNAi construct targeting all three isoforms. Constitutive expression of the hairpin RNA using the 35S CaMV promoter resulted in efficient silencing of all three isoforms in leaves, growing tubers, and sprouting tubers. Neither plant growth nor tuber yield was effected in isoamylase-deficient potato lines. Interestingly, starch metabolism was found to be impaired in a tissue-specific manner. While leaf starch content was unaffected, tuber starch was significantly reduced. The reduction in tuber starch content in the transgenic plants was accompanied by a decrease in starch granules size, an increased sucrose content and decreased hexose levels. Despite the effects on granule size, only little changes in chain length composition of soluble and insoluble glucose polymers were detected. The transgenic tubers displayed an early sprouting phenotype that was accompanied by an increased level of sucrose in parenchyma cells below the outgrowing bud. Since high sucrose levels promote sprouting, we propose that the increased number of small starch granules may cause an accelerated turnover of glucan chains and hence a more rapid synthesis of sucrose. This observation links alterations in starch structure/degradation with developmental processes like meristem activation and sprout outgrowth in potato tubers.

## Introduction

Potato (*Solanum tuberosum*) is a staple food and belongs to the economically most important crops. Tubers formed can store up to 20% of their fresh weight as starch. Starch constitutes a major proportion of the human diet and has also many industrial applications. It is a glucose polymer consisting of two fractions; amylose and amylopectin. Amylose is an essentially linear alpha-1,4-linked polymer, while amylopectin is composed of alpha-1,4-linked chains that are branched via alpha-1,6-bonds resulting in a racemose or tree-like structure [1,2]. The pattern of branch points and the distribution of chain lengths are the primary reasons for the semi-crystalline structure of the starch granules and its insolubility in water. Recent studies in Arabidopsis showed the importance of chain-elongating starch synthases, branching enzymes and debranching enzymes in modifying starch branches to produce the highly-structured starch molecule [3–8].

There are two classes of debranching enzymes in plants; limit dextrinases (LDA) and isoamylases. Isoamylases can be further divided into isoamylase 1 (ISA1), isoamylase 2 (ISA2) and isoamylase 3 (ISA3) sub-classes [9,10]. Although knowledge about LDA is incomplete, in Arabidopsis and maize it seems to be mainly involved in starch degradation. In maize, *lda* null mutants (*zpu1)* are deficient in leaf and seed starch degradation [11] while the role of LDA in Arabidopsis appeared to be partly redundant with ISA3 [12].

In Arabidopsis as well as in potato, ISA1 and ISA2 form a heterodimeric enzyme *in vivo* which is mainly involved in starch biosynthesis [3,9]. Its proposed function is to hydrolyse wrongly-positioned branches refining thereby the structure of the amylopectin molecule. In cereals, there is good evidence that ISA1 can also form an active homomultimetric enzyme complex in addition to the ISA1/ISA2 heteromultimer [1,13]. ISA2 was proposed to be the non-catalytic subunit, since ISA2 proteins from many species carry non-conservative amino acid substitutions in the active site [1]. In Arabidopsis mutants lacking either *ISA1* or *ISA2* the starch structure is altered and glucose chains contain a higher fraction of short branches. Furthermore, these mutants also accumulate the highly branched soluble glucan—phytoglucans [3–6,14].

Similar as in Arabidopsis, potato ISA3 was not found to be strongly associated with the ISA1/ISA2 multimer and was supposed to function as monomer or together with other proteins [9].

Starch degradation is dependent on the accessibility of its constituents, amylopectin and amylose, for the degrading enzymes. Since starch is insoluble, many degrading enzymes do not have access in tissues other than cereal grains, making starch degradation a highly specific and regulated process. There is good evidence that the initial step of starch degradation in Arabidopsis leaf chloroplasts and potato tuber amyloplasts requires the phosphorylation of glucose residues by glucan water dikinases. This disrupts the organised structure making the molecule more soluble and providing degrading enzymes access to the starch granule [15–18]. Although direct evidence is still lacking, it was assumed that in Arabidopsis mutants lacking *ISA1* and/or *ISA2* the rate of starch degradation is accelerated and is less specific due to easier access of degrading enzymes to the soluble phytoglucans molecules [14]. Indirect support for this hypothesis came firstly from the accumulation of starch degradation products during the day in *isa1* and *isa2* mutants and secondly from the fact that phytoglucans were enriched in glucan chains shorter than those produced by the branching enzyme [3]. The third isoamylase isoform, ISA3, is primarily involved in starch degradation, where it hydrolyses branch-points to produce soluble malto-oligosaccharides in the plastid. The enzyme is specific for short branches such as those found in beta-limit-dextrins [9,12,19]. Arabidopsis *isa3* exhibit a starch-excess phenotype [4,6,12]. The glucans released by ISA3 are then further degraded by other starch degrading enzymes, most important of which is beta-amylase [20–22].

Earlier work in potato by Bustos et al. [23] showed that silencing of *ISA1* or *ISA2* led to altered starch formation. While starch content was not significantly altered, potato tubers from these lines accumulated small amounts of soluble glucans and the number of small starch granules increased. Although there was no clear effect on both starch content and structure, soluble glucans contained a slightly higher proportion of shorter chains compared to starch from wild-type tubers and the authors argued that isoamylases are involved in controlling granule initiation as also implied by other studies [24].

The time and rate of potato tuber sprouting is dependent on various factors, one of which is the supply of soluble carbohydrates from the tuber parenchyma to the developing shoot. Initial bud growth does not require massive starch mobilisation, but relies on available soluble hexoses and sucrose (see [25]). After soluble sugars are depleted, carbon supply for the growing sprout is almost exclusively derived from starch degradation, a process which is influenced by the solubility of starch, as explained above. Despite the clear role which starch degradation may play in sprout outgrowth, knowledge of the precise mechanisms and enzymes involved is currently lacking. Here, we report on the simultaneous silencing of *ISA1*, *ISA2* and *ISA3* in transgenic potato plants using one chimeric RNAi construct. At harvest, tubers of the transgenic lines contained a reduced amount of starch which was paralleled by higher sucrose levels. These changes in carbohydrate accumulation were accompanied by an early sprouting phenotype. Closer inspection of the starch granules revealed an increased number of small starch granules and a higher percentage of soluble glucans. This occurred in the absence of clear changes to the branch structure of either soluble or insoluble glucans.

## Materials and methods

### Plant material, growth conditions and sampling

*Solanum tuberosum* L. cv. Solara and transgenic lines triple-ISA-7, -16 and -39 were maintained in tissue culture on MS medium [26] containing 2% (w/v) sucrose with a 16 h light and 8 h dark period. After propagation, plants were transferred into the greenhouse and were cultivated in individual 4 l pots at 50% humidity with 16 h of supplemental light (150 μmol quanta m^-2^ s^-1^) and 8 h of darkness. The temperature regime followed the light/dark cycle with 21°C and 18°C, respectively. Leaf samples were taken from fully expanded source leaves using a cork borer (#4). Tubers were harvested after 12 weeks and stored in darkness at room temperature. Tuber samples from freshly harvested or stored tubers were taken by punching a cork borer #4 through the middle of equally sized tubers and rasping the cylinder into ca. 1.5 mm thick slices. For RNA 8 slices were taken, whereas for determination of sugars 1–2 slides were taken, flash frozen in liquid nitrogen and stored at -80°C until extraction. Samples from the sprout-associated tuber parenchyma tissue were taken by means of a cork borer #2. The tissue approximately 5 mm beneath the bud was cut into 1.5 mm slices and 2 slices were deep frozen until analysis.

### RNAi construct design and potato transformation

To generate the RNAi construct targeting the three different *isoamylase* (*ISA*) genes, individual PCR reactions for each gene were conducted using *ISA1*-specific (5´- CAC C CT CGT GGA ATG CTG TAA ATG -3´ and 5´- GTC ACT CCC CAT GCC AAC TTG GTA AAG GC -3´), *ISA2*-specific (5´- GTT GGC ATG GGG AGT GAC CAA TCT CCT CC-3´ and 5´-GTC ACT CCC CAT GCC AAC TTG GTA AAG GC -3´) and *ISA3*-specific (5´- GCA GCT GAG C CAA TCT CTG AAT CAC CAG CAC C-3´ and 5´- GCA AAG AAC ACT AGC AAG ATC -3´) primers (NCBI accession numbers AY132996, AY132997, AY132998). Primers contained additional nucleotides at either the 3´ of 5´end allowing the different PCR fragments to hybridise in a subsequent PCR reaction. This produced a single DNA fragment used for sub-cloning into the entry vector pENTR-D-TOPO, followed by insertion into the vector pK7GWIWG2(II) (Invitrogen) using gateway technology. The construct obtained was used for potato transformation as described previously [27].

### Quantitative real-time PCR

Quantitative real-time PCR was performed as described previously [28] using specific primer sets for *ISA1* (5´-GGC AAA TGG AGA GGA CAA CA -3´ and 5´- ATG GGA ACA CCT TGG GAA AC -3´), *ISA2* (5´- TTA TCC TTC CGC CAC CTC -3´ and 5´- CTT CAA CTG GAG TTC CCT TCT-3´) and *ISA3* (5´- GAC GCT TGC CCT TCA TTC -3´and 5´- CTC CTG TGC GGT TCT TCT GT -3´). Expression of ubiquitin (*ubi3*, L22576) was used for normalization. Relative expression of target genes was calculated according to Pfaffl (2001) [29].

### Metabolite measurements

Measurement of starch and soluble sugars were performed as described previously [30]. For soluble glucan measurement, tuber material was homogenised in geno grinder (SPEX). The material was kept frozen at all times and to around 600 mg material 3 ml of ice-cold 1.12 M perchloric acid was added and mixed well. 2.7 ml of suspension was transferred to a fresh tube and centrifuged for 10 min (3000*g*) at 4°C. The supernatant (2.2 ml) was transferred to a fresh tube, kept on ice, and subsequently neutralised using neutralisation buffer (2 M potassium hydroxide, 0.4 M MES buffer and 0.4 M potassium chloride) and centrifuged for 10 min (3000 g) at 4°C. The supernatant was transferred to a fresh tube and used to determine soluble glucan content. Soluble glucans were determined by measuring the amount of glucose released by treatment with α-amylase and amyloglucosidase [31].

### Granule size distribution measurements

Starch grains from 3 individual tubers of each genotype were extracted, stained with iodine and individually analysed by light microscopy. Micrographs of iodine stained starch granules were taken (20 x magnification) and the surface area of individual granules calculated using imaging and segmentation software ImageJ (http://rsbweb.nih.gov/ij). A detailed protocol on how to determine the area of two dimensional structures using ImageJ software is available online (naranja.umh.es/~atg/tutorials/VGIV-MeasuringCellsImageJ.pdf). On average, for each replicate 160 starch granules were measured and the size distribution was calculated based on total numbers counted in each replicate.

### Soluble and insoluble glucan structure analysis

Soluble and insoluble glucan structure analysis was performed as described previously [6].

## Results and discussion

### A chimeric RNAi construct allows the simultaneous silencing of *ISA1*, *ISA2* and *ISA3* in transgenic potato plants

Although RNAi silencing of genes in plants is done routinely, only few reports targeting multiple members of one gene family by one chimeric RNAi construct have been reported. In rice, Miki et al. [32] designed one chimeric RNAi construct to simultaneously silence four members of the rice *Rac* gene family. In barley, Carciofi et al. [33] produced amylose-only starch by silencing all starch branching enzymes using one chimeric RNAi hairpin construct. In potato, silencing of *Sbe1* and *Sbe2* was achieved by co-transformation of two gene-specific RNAi constructs [34], while reduced activities of SSII and SSIII were obtained by transforming an chimeric construct [35].

**Fig 1:**
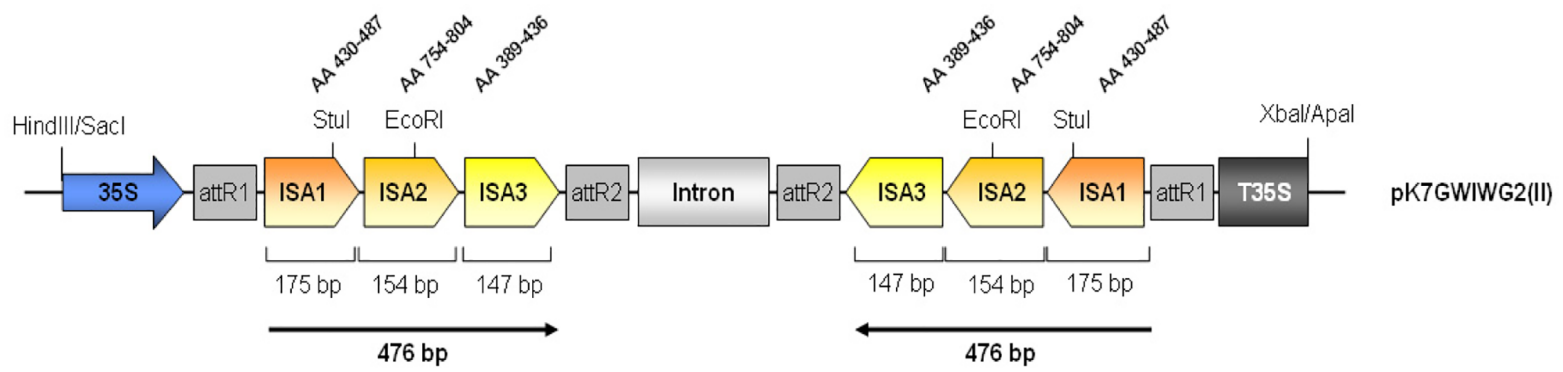
Schematic representation of the chimeric RNAi hairpin construct.

Here we report on the simultaneous silencing of three *ISA* genes in potato. Their sequences show 44% to 52% nucleotide sequence identity between each other. Since the overall sequence homology is rather low and there are no stretches longer than 15 nucleotides of sequence identity, we decided to create a chimeric RNAi construct to achieve simultaneous silencing of all three *ISA* genes (Fig 1). Gene-specific fragments were amplified by PCR and purified. Fusion of the three gene fragments was accomplished by overlap PCR. This chimeric fragment was cloned into the gateway vector pK7GWIWG2(II) between the cauliflower mosaic virus (CaMV) 35S promoter and terminator to drive constitutive expression of the hairpin construct (Fig 1).

The construct was transformed into potato and 51 transgenic lines were regenerated. Initially, northern blot hybridisation was used to screen for transgenic potato lines with simultaneously reduced accumulation of *ISA1*, *ISA2* and *ISA3* transcripts. Pre-screening of 40 independent transgenic lines revealed 13 lines, showing reduced *ISA* mRNA levels in leaves of greenhouse-grown plants. These lines were tested in an independent experiment, which confirmed efficient silencing of all three *ISA* genes in 12 cases (S1A Fig). Based on the degree of silencing in leaves and the reduced tuber starch content (S1B Fig), three lines (7, 16 and 39) were selected for subsequent studies.

**Fig 2:**
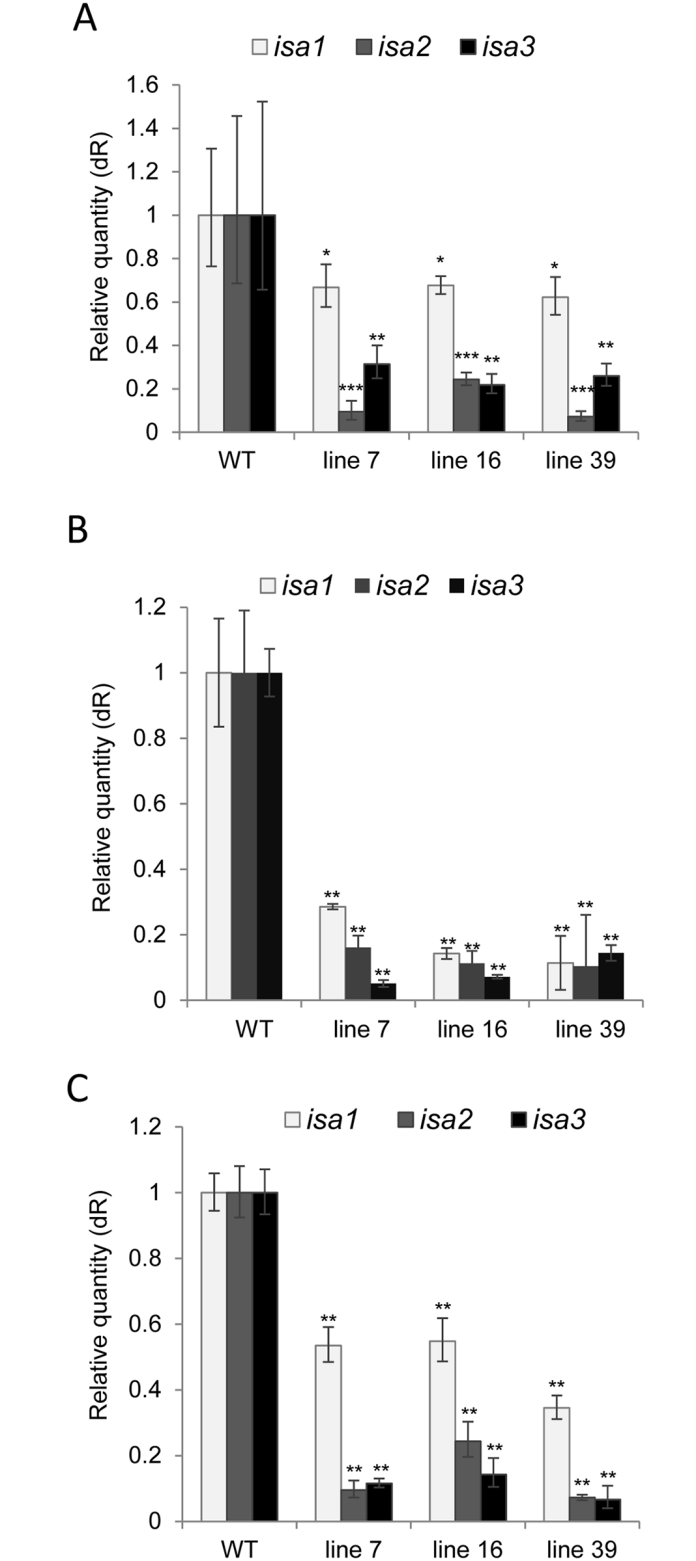
Relative expression of isoamylase isogenes in wild type (WT) and selected transgenic lines 7, 16 and 39 as determined by qRT-PCR. Relative expression of *ISA1*, *ISA2* and *ISA3* were determined in (A) source leaves, (B) mature potato tubers, (C) in sprouting tubers. Values were normalized to *Ubiquitin* and displayed relative to the expression level of the wild type which was set to one. Values displayed are the mean +/- SD of three to four independent biological samples each with two technical replicates. Significant differences to wild type were calculated using a two-tailed t-test assuming equal variances and are indicated by asterisks (***p<0.001; ** p< 0.01, *p<0.05).

These lines were amplified together with wild-type plants in tissue culture and then transferred into the greenhouse for further analyses. To verify silencing of all three *ISA* genes in these lines, leaf and tuber samples were taken and subjected to a quantitative real-time PCR (qPCR). Transcript accumulation of all three genes was significantly reduced in these lines in leaves (Fig 2A) as well as in mature (Fig 2B) and sprouting tubers (Fig 2C) as compared to the untransformed controls. Interestingly, silencing of all isoforms was most efficient in mature, freshly harvested tubers with rates of inhibition between 72–89% (*ISA1*), 84–90% (*ISA2*) and 86–95% (*ISA3*) in the selected lines, respectively. In particular, *ISA1* was less efficiently silenced in leaves and sprouting tubers (Fig 2). Based on sequence identity, cross silencing of the individual genes is unlikely. However, altered expression of one gene may influence expression levels of the others. In this context, it has also been shown that silencing of either *ISA1* or *ISA2* in potato leads to the reduction in expression of the other gene indicating that the transcript amount of these two genes may be coordinated [23]. It is doubtful, however, that a similar mechanism would exist for *ISA3*, since it presumably functions independently from the other two proteins, which form a heteromultimeric enzyme together.

In any case, expression of the chimeric hairpin construct allowed the regeneration of transgenic potato plants with significantly reduced accumulation of *ISA1*, *ISA2* and *ISA3*-specific mRNAs in leaf and tuber tissues.

### Silencing of *ISA1*, *ISA2* and *ISA3* does not affect leaf starch content but significantly alters starch accumulation in potato tubers

**Fig 3:**
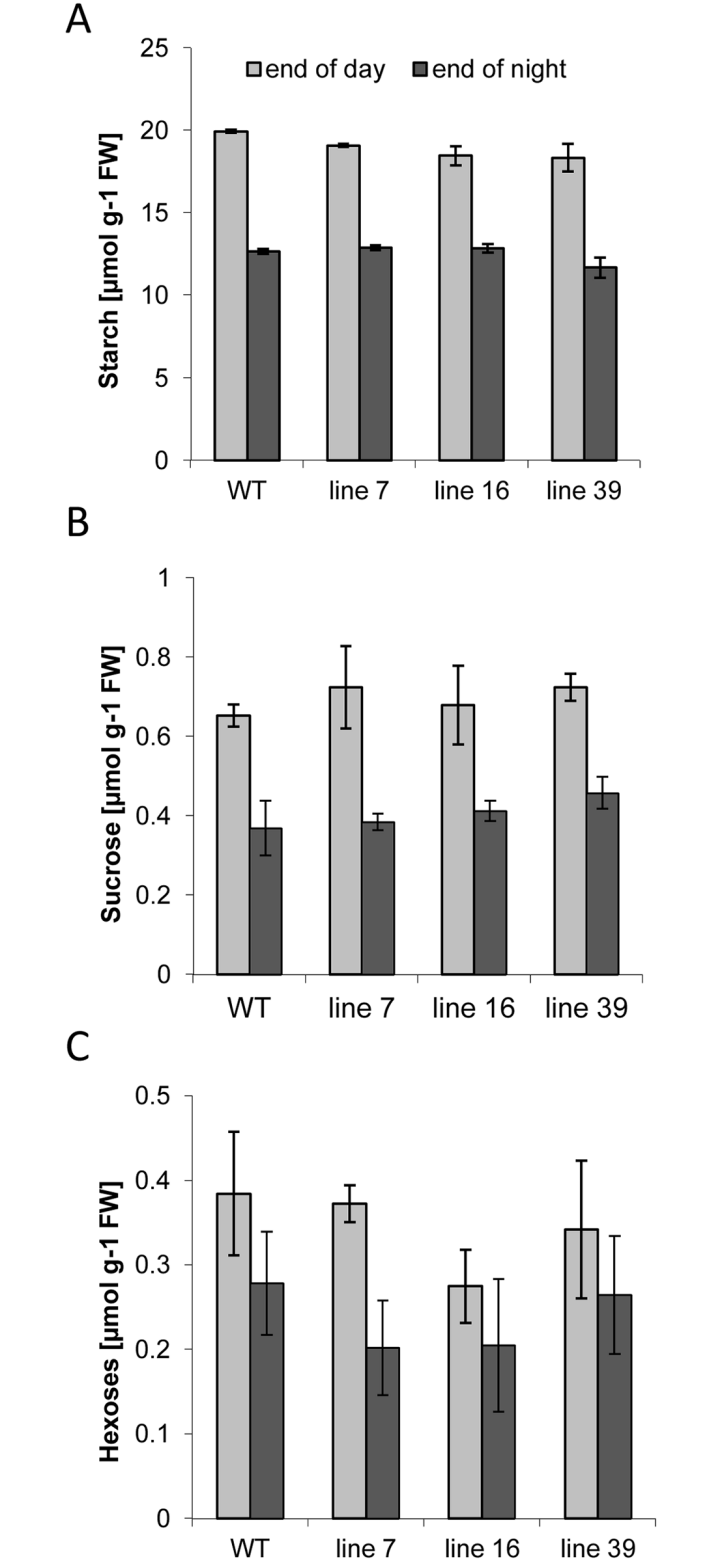
Impact of reduced expression of *ISA* isogenes on soluble sugars and starch content in source leaves. Metabolite contents in wild type and *ISA*- silenced plants were determined at the end of the light period (after 16h light) (light grey) and at the end of the dark period (after 8h darkness) (dark grey). A) starch B) sucrose, C) hexoses. Values represent the mean +/- SE of 5 biological and 2 technical replicates each.

There were no visible phenotypical changes to the aerial parts of the plants. In order to investigate whether *ISA* silencing affects accumulation of starch in leaves, samples from fully expanded source leaves were taken at the end of the day (after 16 h of light) and at the end of the night period (after 8 h darkness) and the amount of soluble sugars and starch was determined. As shown in Fig 3, leaf starch content was not significantly changed at both time points (Fig 3A). Similarly, there were no changes in the levels of sucrose and hexoses in leaves of transgenic lines as compared to controls (Fig 3B and 3C). The failure to obtain significant changes in carbohydrate accumulation in leaves may be caused by a less efficient silencing of the target genes. Additionally, overall expression of all three isoamylase genes in leaves is lower as compared to tubers [36]. Therefore, it is possible that the remaining mRNAs results in sufficient isoamylase activities.

**Fig 4:**
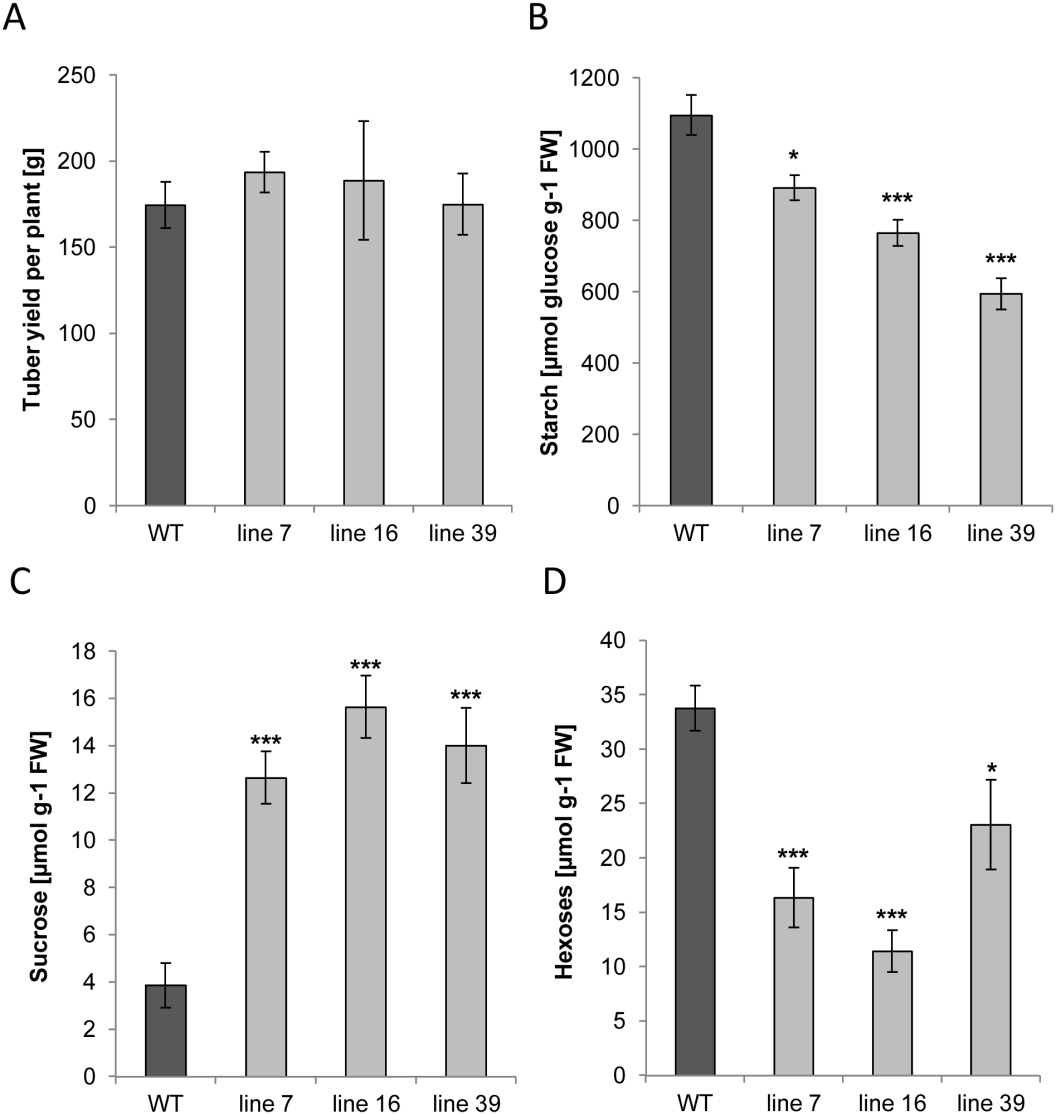
Effect of reduced *ISA* gene expression on tuber yield and content of sugars. Parameters were determined from greenhouse-grown plants at harvest A) Tuber yield was determined from 5 plants each. B) starch content, C) sucrose content and D) hexose levels were determines from tuber parenchyma. Values represent the mean +/- SE of 5 biological and 2 technical replicates Significant differences to wild type were calculated using two-tailed t-test assuming equal variances and are indicated by asterisks (***p<0.001, *p<0.05).

To analyse the impact of *ISA* silencing in tubers, growing tubers were harvested and the impact on tuber yield and sugar content was determined. There was no change in total tuber fresh weight per plant (Fig 4A). However, there was a significant reduction in tuber starch content in mature tubers harvested from the transgenic lines (Fig 4B). The strongest reduction was detected in line 39 accumulating only about half of the starch of wild-type tubers. This confirms our results from the screening (S1B Fig) and may be caused by the overall strongest *ISA* transcript inhibition detected in this line (Fig 2). The decreased starch content was paralleled by a decreased dry weight to fresh weight ratio which was reduced by 10% to 22% in the transgenic lines relative to that of wild type tubers (S2 Fig). Moreover, the transgenic tubers exhibited an increased sucrose (Fig 4C) and a decreased hexose (Fig 4D) content as compared to the wild-type controls. Silencing *ISA1* and *ISA2* in potato plants, Bustos et al. (2004) did only find moderate changes in the overall tuber starch content [23]. The discrepancy may be explained by either different degrees of silencing, different genotypes used or the simultaneous silencing of all three *ISA* isoforms in this study.

### Starch granule size is strongly reduced in ISA-deficient potato tubers

**Fig 5:**
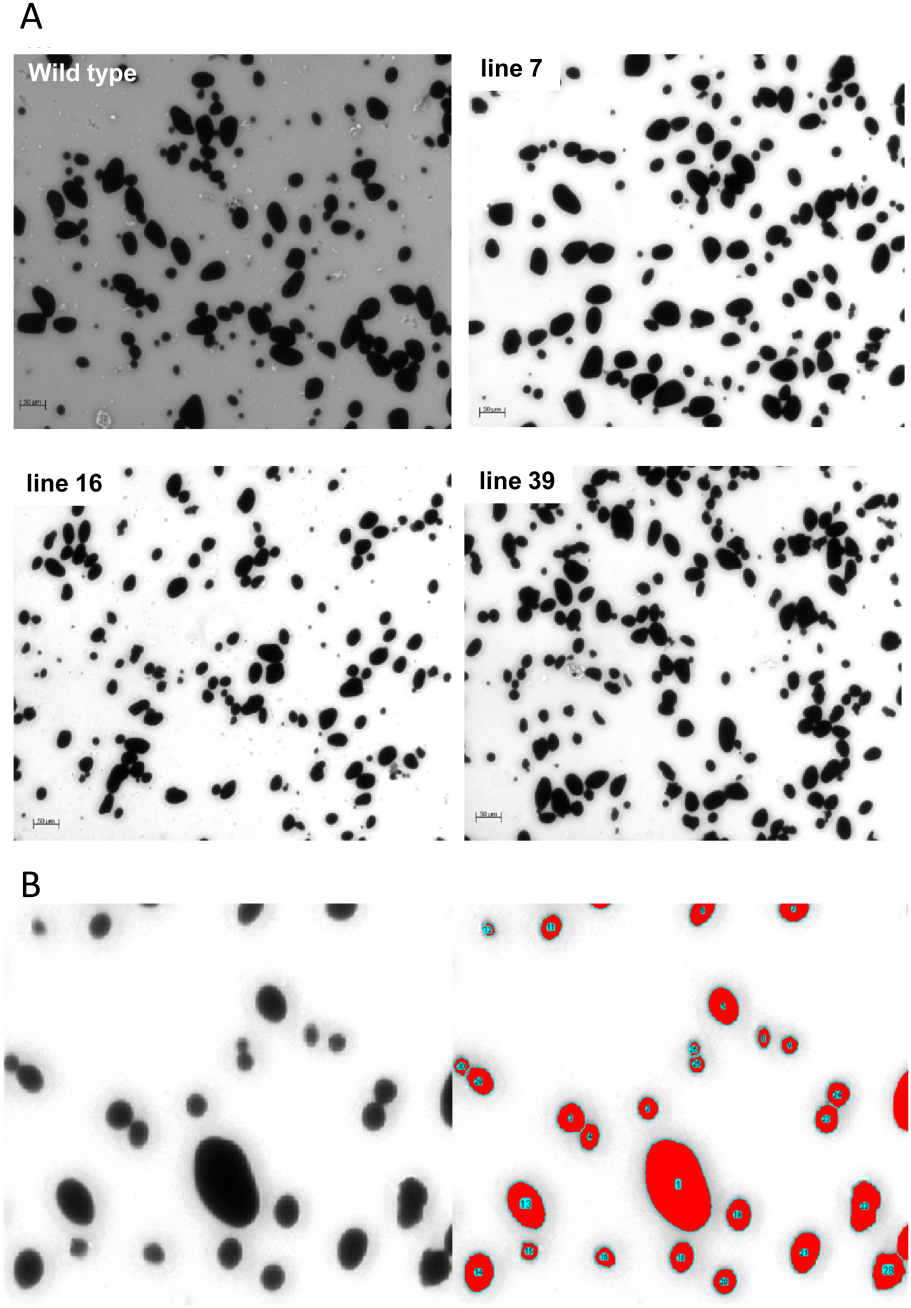
Determination of the starch granule size. Starch granules from freshly harvested tubers were extracted, stained with iodine and inspected under the microscope. A) representative micrographs of iodine-stained starch granules derived from tubers of wild-type and *ISA*-silenced lines 7, 16 and 39. B) Representative image illustrating the segmentation of images by means of the ImageJ software tool to determine the relative size of each 2D starch granule. The software allows for the manual separation of granules connected to each other in the image.

**Fig 6:**
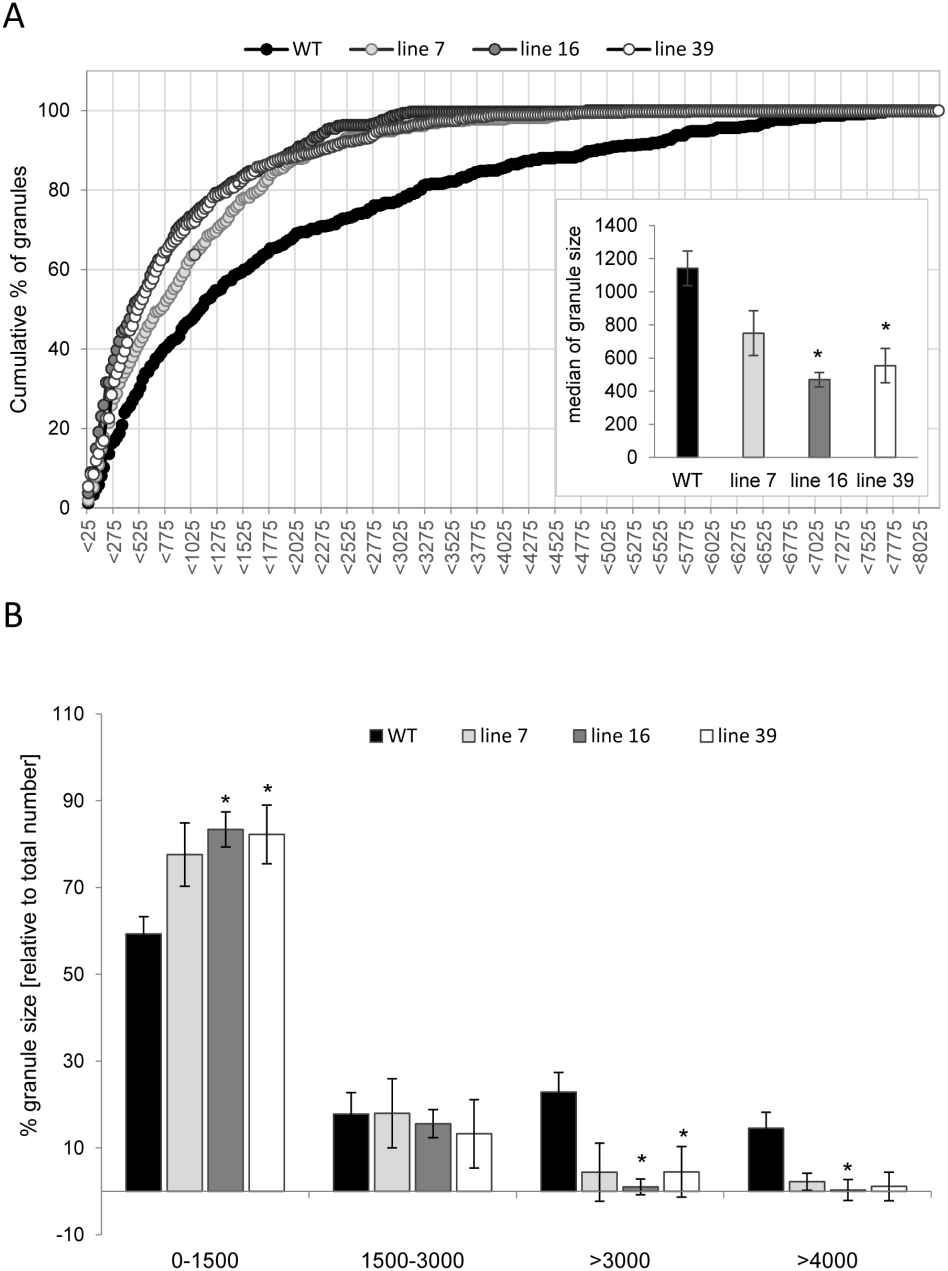
Starch granule size distribution. Size distribution was determined as pixels’ size from ca. 150 starch granules of each replicate using ImageJ software as schematically shown in Fig 5. A) Granules were compiled into groups and the number of granules within a specific range (e.g. >25, >50, >75, etc.) were counted, calculated as relative percentage of total number and values cumulatively plotted. The inset shows the median granule size of each genotype. B) Bar charts of selected groups of relative starch granule sizes subjected to t-test test analysis. Values represent means +/- SE from 3 individual tubers (n = 3). Significant differences to wild type are indicated by asterisks (*p<0.05). Line 7 (light grey), line 16 (dark grey), line 39 (white) wild-type control (WT; black).

Although, the tuber starch content of *ISA1*/*ISA2* silenced potato plants was almost unchanged, granule size and number significantly changed [23]. Transgenic tubers contained more, but smaller granules. This led the authors to conclude that ISA1 and ISA2 suppress initiation of glucan molecules [23]. To investigate the influence of the triple silencing approach on starch structure, we aimed to quantify granule size. Thus, starch granules from freshly harvested tubers were isolated, stained with iodine and viewed under a microscope (Fig 5). Microscope pictures were taken and the relative 2D granule size based on pixel counts was determined using ImageJ software (http://rsbweb.nih.gov/ij). This revealed significant changes in starch granule size, with transgenic lines accumulating relatively higher amounts of small granules than wild-type (Fig 6A). This was especially evident for the relative granule sizes smaller than 1500 and larger than 3000 which were significantly higher or smaller in lines 16 and 39 (Fig 6B), respectively, but became also clear from the median granule size (Fig 6A). This observation is in agreement with the previous publication on *ISA1* and *ISA2* silenced potato plants [23].

### ISA-deficient potato tubers are characterized by an early sprouting phenotype

**Fig 7:**
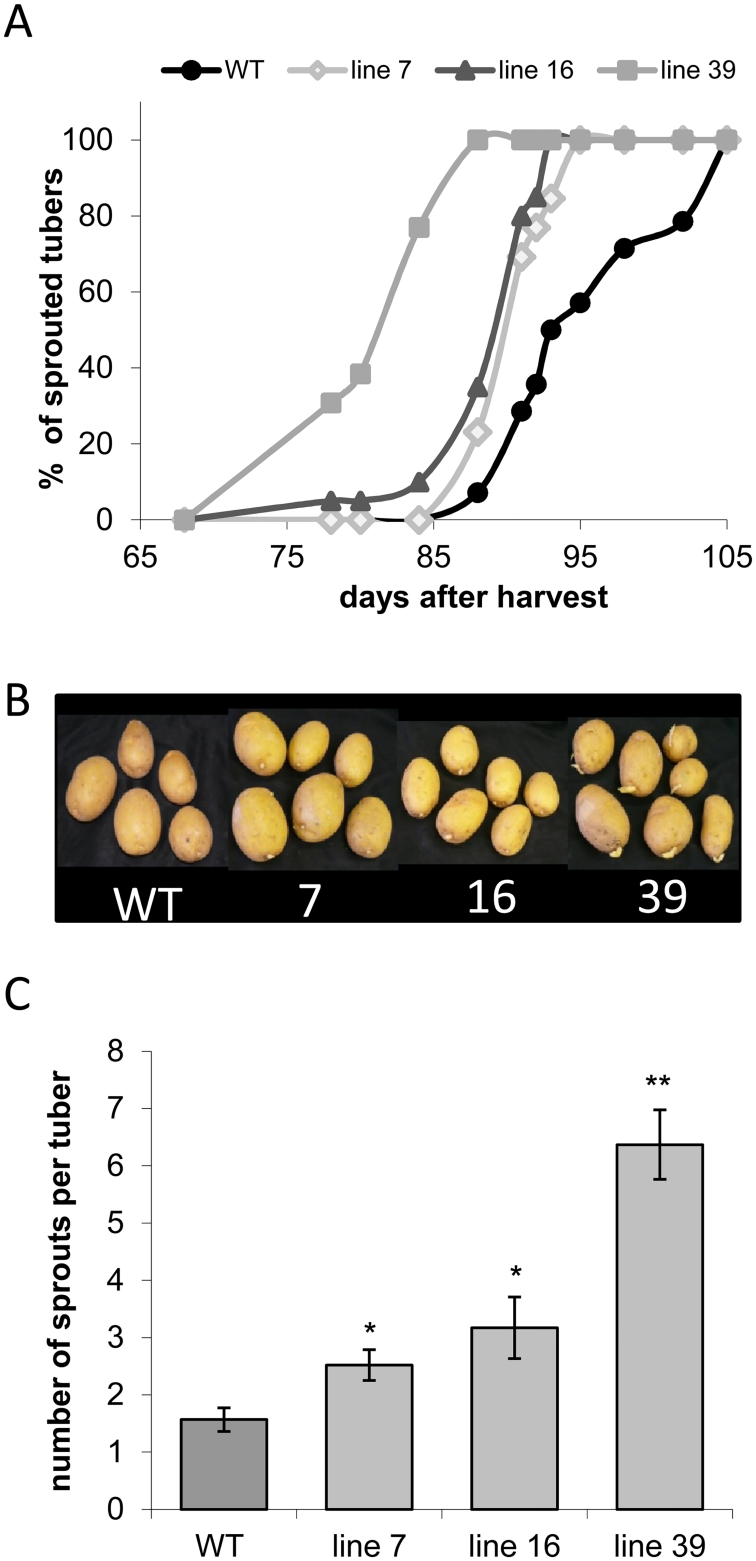
Impact of *ISA* silencing on tuber sprouting. Tubers of 5 plants each from wild type (WT) and transgenic lines 7, 16 and 39 were stored after harvest at room temperature in darkness. A) Sprouting kinetics. To monitor the impact on dormancy length, 2–5 similar sized tubers from each plant were picked (n = 13–20) and their sprouting behaviour was regularly scored over a 15-week period until 100% sprouting had been reached in wild-type tubers. A tuber was considered to sprout when sprouts of 2 mm length became visible. B) Photographs of transgenic (lines 7, 16, 39) and control tubers taken after 13 weeks of storage showing that the transgenic lines sprout earlier than the wild-type controls (WT). C) Number of sprouts per tuber. Number of sprouts formed per tuber were counted from 13–20 individual tubers. Values represent the mean +/- SE. Significant differences to wild type were calculated using two-tailed t-test assuming equal variances and are indicated by asterisks (**p<0.01, *p<0.05).

One of the most striking characteristics of the transgenic plants was the reduced dormancy period. *ISA* silencing led to a change in the onset of sprouting, with the transgenic lines sprouting earlier than the control plants (Fig 7A and 7B). In the strongest line 39, visible sprouts appeared already after sixty-eight days and 100% sprouting was reached on day eighty-five (Fig 7A). The other transgenic lines showed visible sprouting between days seventy-eight and eighty-five, with 100% sprouting reached after ninety-three days in the weakest line, line 7. First sprouts of wild-type tubers could be detected after eighty-eight days and 100% sprouting was reached after one hundred and three days (Fig 7A). This shows that wild-type tubers started sprouting almost 3 weeks later that those of line 39.

Sprouting behaviour of the transgenic tubers was also changed in terms of the number of sprouts produced per tuber. As shown in Fig 7C the transgenic tubers had significantly more sprouts per tuber than the control tubers.

**Fig 8:**
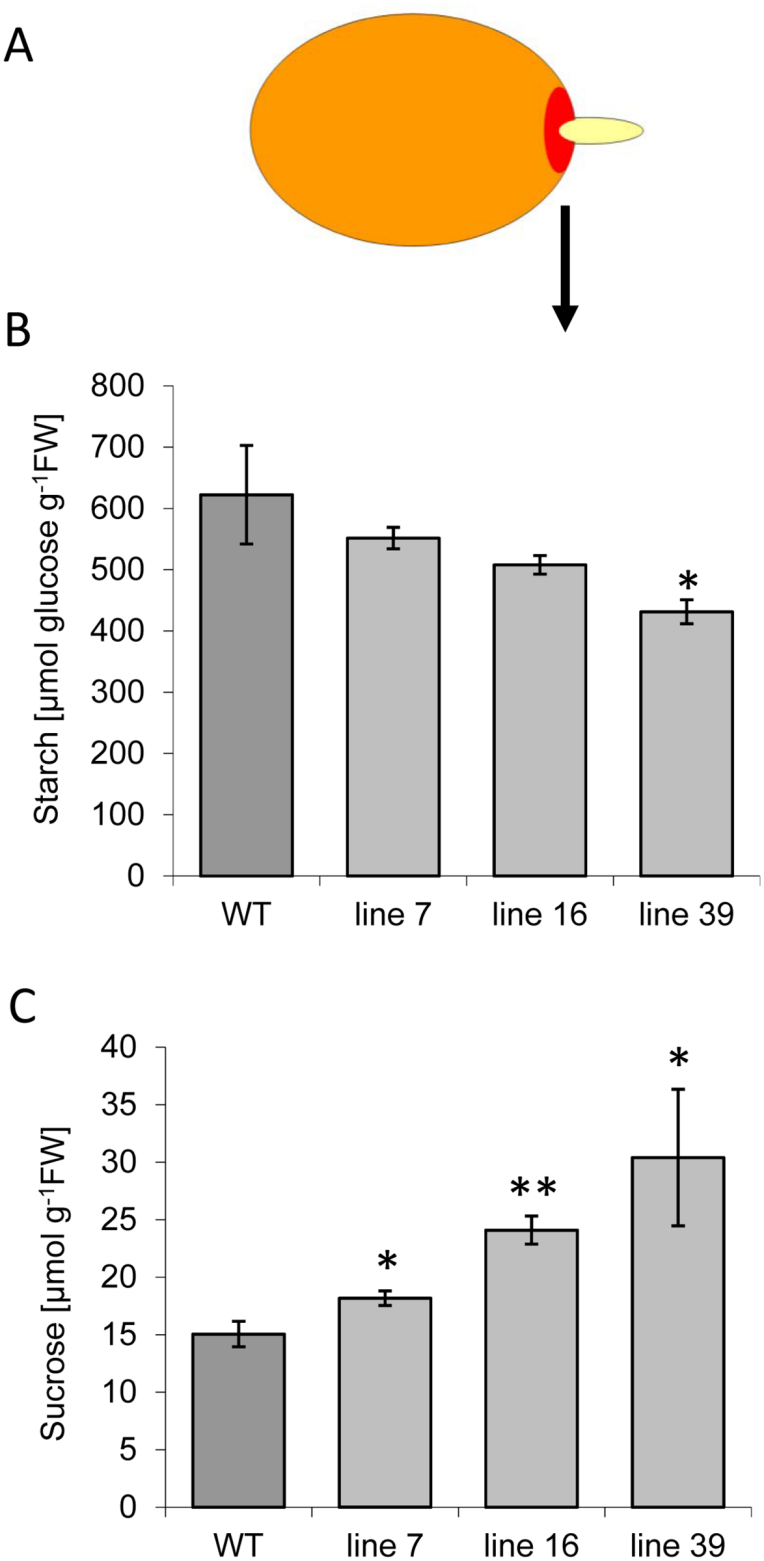
Contents of sucrose and starch in tuber parenchyma tissue associated with the sprout. Samples from wild-type and transgenic tubers were taken after 15 weeks of storage, when sprouts became visible in wild-type tubers. A) Schematic representation of a potato tuber to illustrate the sampling. A cork borer (# 2) was used to isolate the tissue approximately 5 mm below the bud indicated in red. It can be assumed that this region is enriched in vascular tissue. B) Starch content and C) sucrose content in the tissue below the growing sprout. Values represent the mean +/- SE of 5 independent tubers. Significant differences to wild type were calculated using two-tailed t-test assuming equal variances and are indicated by asterisks (**p<0.01, *p<0.05).

Potato tuber sprouting is controlled by several endogeneous and environmental factors. Amongst the endogenous factors, availability of sucrose plays a central role [25]. During dormancy, sucrose levels rise especially in tissues below sprouting buds. This increase in sucrose levels is not accompanied by large changes in starch content of parenchyma cells. Changes in starch content can only be detected in tissues directly beneath the outgrowing bud. To test the hypothesis that the observed sprouting phenotype is a consequence of high sucrose levels, the starch and sucrose content in the tissue beneath the sprouting apical bud was analysed. Samples were taken at the time-point when sprouts became visible in the wild-type tubers as illustrated in Fig 8A. Notably, the reduction in the starch content in the transgenic lines was less apparent in this tissue as compared to developing tubers, with only line 39 having significantly less starch (Fig 8B). However, this result could be deceptive, since starch content was calculated on a fresh weight basis. As indicated in S2 Fig, transgenic tubers contain significantly more water at harvest, as compared to the control. Hence, it cannot be excluded that transgenic tubers evaporate more water during storage, leading to a relative increase in the starch content as calculated on a fresh weight basis. Nevertheless, the sucrose content of transgenic lines was much higher as compared to the wild-type controls (Fig 8C). This finding is in agreement with the hypothesis that the reduced dormancy periode of *ISA*-silenced potato tubers is mediated by the accumulation of sucrose. One possible explanation for the observed sucrose accumulation is that due to ISA inhibition starch granule formation is impaired, a large number of small granules accumulate and their surface may be easier accessible for starch degrading enzymes.

This assumption may be counterintuitive since also *ISA3* mRNA levels were strongly decreased in the transgenic lines. ISA3 was shown to be an important starch degrading enzyme [3,4,6], causing reduced starch degradation in Arabidopsis leaves when mutated. Likewise, in potato, ISA3 preferentially uses beta-limit dextrins as substrate arguing for its role as debranching enzyme during starch degradation [9].

As shown in Fig 6 tubers harvested from *ISA*-silenced lines accumulated smaller starch granules that may exhibit a less organised structure and an increased solubility. This could positively affect starch degradation during storage. Thus, one possible explanation for the early sprouting phenotype is an impaired starch biosynthesis caused by a reduced expression of *ISA1*/ *ISA2* and therefore a stimulated sucrose biosynthesis. This is in agreement with work form Bustos et al. 2004 [23] who concluded from analysis of ISA1 and ISA2 antisense potato tubers that the heteromeric complex controls granule initiation. However, the authors did not report on an altered sprouting behaviour of the transgenic tubers.

**Fig 9:**
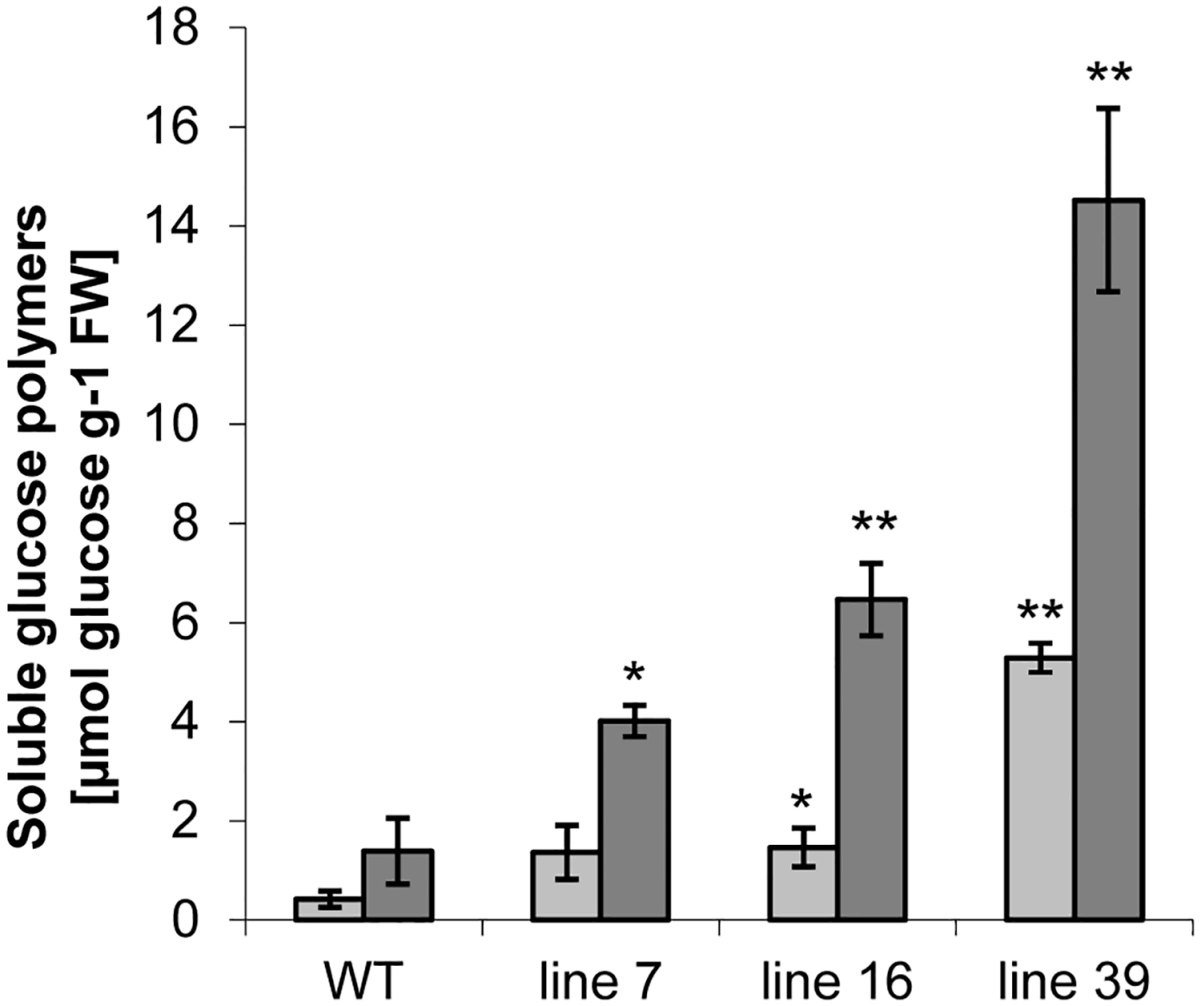
Content of soluble glucans (phytoglycogen) in tubers. The amount was determined at harvest (light grey) and after 4 months of storage in the parenchyma around growing sprout (dark grey). Error bars represent standard errors (n = 4–5). Asterisks indicate statistically significant differences (* p < 0.05 and ** p < 0.01).

Next, we examined whether transgenic tubers contained an elevated amount of soluble glucose polymers (phytoglucans). Here, samples from freshly harvested tubers and from the parenchyma tissue associated with the tuber sprouts were analysed. In all samples a 3-fold increase in the content of soluble glucans was measured during tuber storage (Fig 9). However, the soluble glucan content was 3- to 10- fold higher in the transgenic as in control tubers at both harvest and the onset of sprouting with the strongest increase observed again in line 39 (Fig 9). Hence, the elevated levels of soluble glucans are indicative for an altered starch composition and suggest an increased accessibility of starch for degradation which concurs with the earlier sprouting.

### Glucan structure is only slightly altered in *ISA*-silenced potato tubers

**Fig 10:**
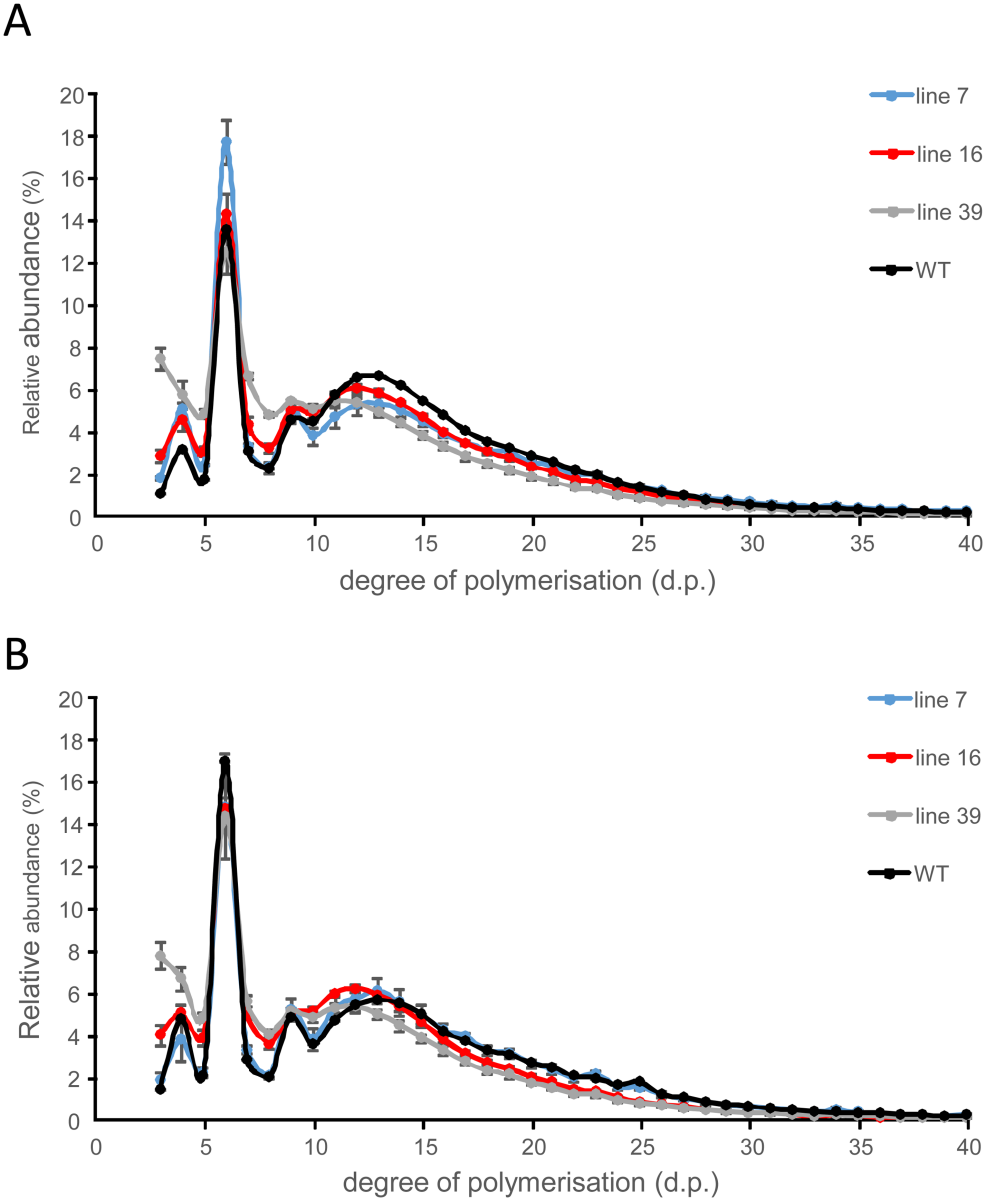
Structure of soluble glucan polymers determined as relative chain length distribution. A) in sprout-associated tuber parenchyma and B) in tuber parenchyma not associated with a sprout. Line 7 (blue), line 16 (red), line 39 (grey) and the wild-type control (WT; black). Values represent mean +/- SE of 3–4 biological replicates (n = 3–4).

**Fig 11:**
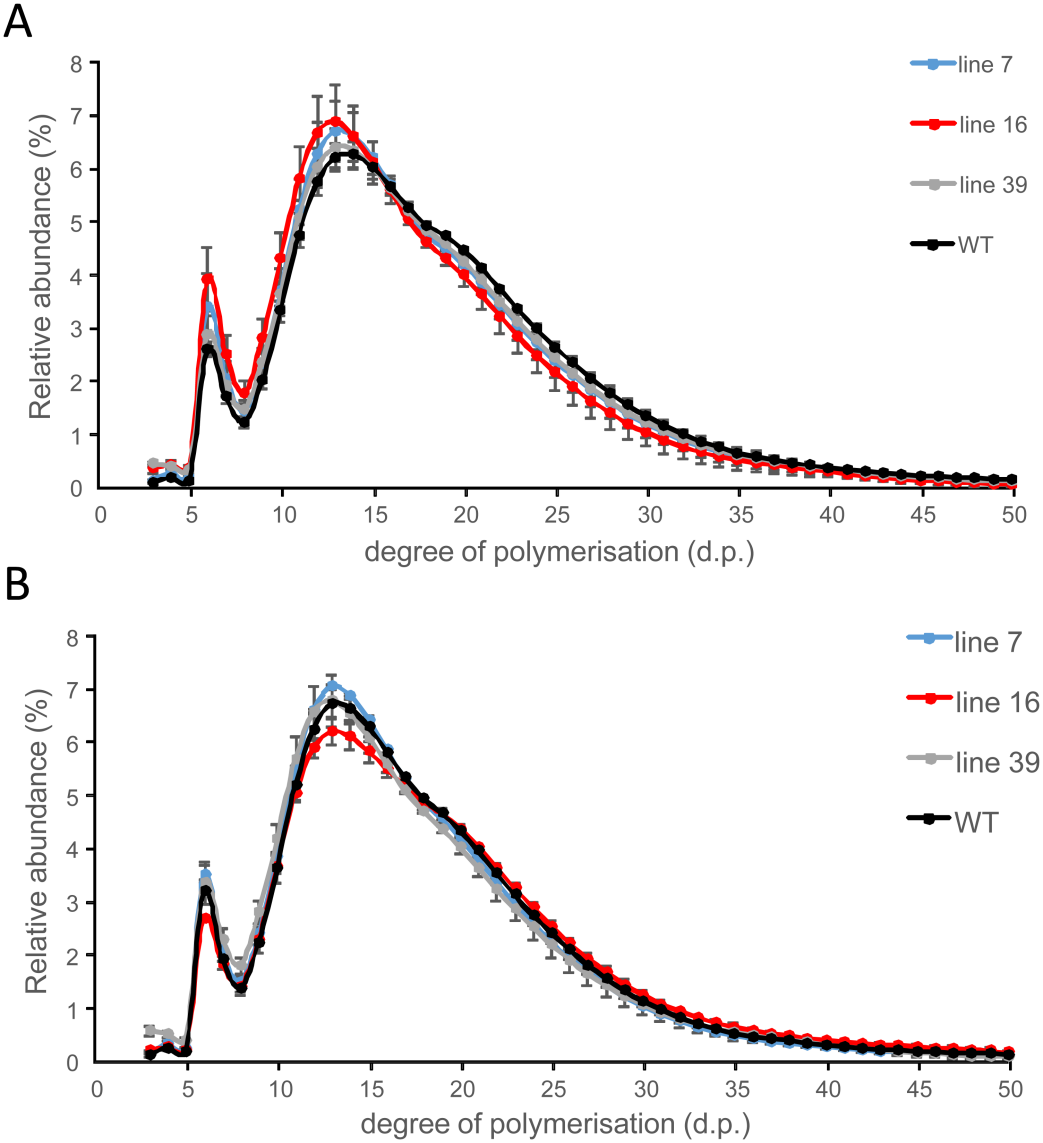
Starch structure determined as relative chain length distribution. Starch from sprouting tubers was either isolated from parenchyma associated with the sprout (A) or (B) from parenchyma not associated with the sprout. Line 7 (blue), line 16 (red), line 39 (grey) and the wild-type control (WT; black). Values represent mean +/- SE of 3–4 biological replicates.

In order to investigate whether the changes in granule size distribution were accompanied by alterations in the glucan structure, the chain length distributions of soluble and of insoluble glucan fractions were determined in sprouting tubers (Figs 10 and 11). Structural analysis of the soluble glucans was complicated by the low amounts present in the tubers, particularly in the controls. This meant that the control samples had to be pooled for the measurements and consequently no statistical tests could be performed. Furthermore, a minor contaminant in the reagents used was visible on the chromatograms, co-migrating with chains with a degree of polymerization (DP) of 6. This rendered DP6 impossible to quantify accurately in these samples. Despite these analytical limitations, the data suggest that the structure of the soluble glucans was affected in lines 16 and 39. Particularly in line 39, there was an increase in the relative abundance of shorter chains (DP 3, 4, 5, 7 and 8) in samples taken at the time of sprouting in both the parenchyma below the sprout (Fig 10A), and in parenchyma not associated with the sprout (Fig 10B).

In contrast to soluble glucans, large amounts of insoluble glucans (starch) were extracted for all genotypes. Here, no significant differences in the composition of these insoluble glucans could be detected at any of the time points or tissues analysed (Fig 11).

However, our data indicate that the transgenic lines form a higher percentage of small granules and possess a higher amount of soluble glucans which tend to have a lower DP. This was most obvious in line 39 which is in accordance with the phenotypes and physiological data presented.

Hence, the reduced starch content of transgenic tubers together with the lower granule size can be interpreted that there were changes in the starch granule structure at the macromolecular level (e.g. less dense packing of amylopectin, leading to increased water content of the starch granules). Moreover, our data indicate that these changes may primarily be caused by altered expression of *ISA1/ ISA2*. Furthermore, since these studies were performed on lines which had only reduced expression of the target genes and were not null mutants, it can be expected that changes occurred non-homogenously, resulting in starch granules with varying structure.

## Conclusions

Using a chimeric hairpin construct we were able to generate transgenic potato plants accumulating significant less *ISA1*, *ISA2* and *ISA3* transcripts. Despite constitutive expression of the construct, only tuber starch metabolism was impaired in transgenic potato plants. Tubers of silenced plants accumulated less starch and hexoses, but more sucrose as compared to the untransformed control. We propose that due to reduced activity of ISA1/ISA2, but not the complete absence, changes to the glucan and granule structure occurred. This would lead to certain parts of the granule being more soluble and accessible to the degrading enzymes, leading to the production of sufficient amounts of sucrose during storage to initiate early sprouting. High sucrose levels may also explain the increased number of growing sprouts per tuber. This assumption is supported by studies in *Pisum sativum*, which showed that apical dominance is initially regulated sugar availability and that artificially increased sucrose levels result in bud release [37].

## Supporting information

S1 Fig Screening of transgenic potato plants expressing the triple ISA RNAi construct. A) Analysis of transgenic lines by northern blotting. Total RNA was extracted from 13 pre-selecetd transgenic lines. Twenty μg of total RNA was separated in a formaldehyde-containing agarose gel and blotted onto nitrocellulose membrane. The membrane was consecutively probed with *ISA1*, *ISA2* and *ISA3* specific ^[32]^P-labelled probes and a with 18S rRNA probe as loading control. B) Tuber starch content in transgenic lines. Two replicates were taken from 5 tubers per line after harvest. Values are the mean +/- SE. Asterisks indicate statistically significant differences at 5% level (* p < 0.05).
(TIF)

S2 Fig Dry weight to fresh weight ratio of tubers. Slices were taken from 5 tubers per line and the fresh weight was determined. Subsequently, slices were dried in an oven at ca. 60°C for 2 days and weighed. Values are the mean +/- SE. Asterisks indicate statistically significant differences at 5% level (* p < 0.05).
(TIF)
