# Supplementary figures and images for: Simultaneous silencing of isoamylases ISA1, ISA2 and ISA3 by multi-target RNAi in potato tubers leads to decreased starch content and an early sprouting phenotype
Source: PLoS One. 2017 Jul 14;12(7):e0181444. doi: 10.1371/journal.pone.0181444 (PMC5510849; doi:10.1371/journal.pone.0181444)

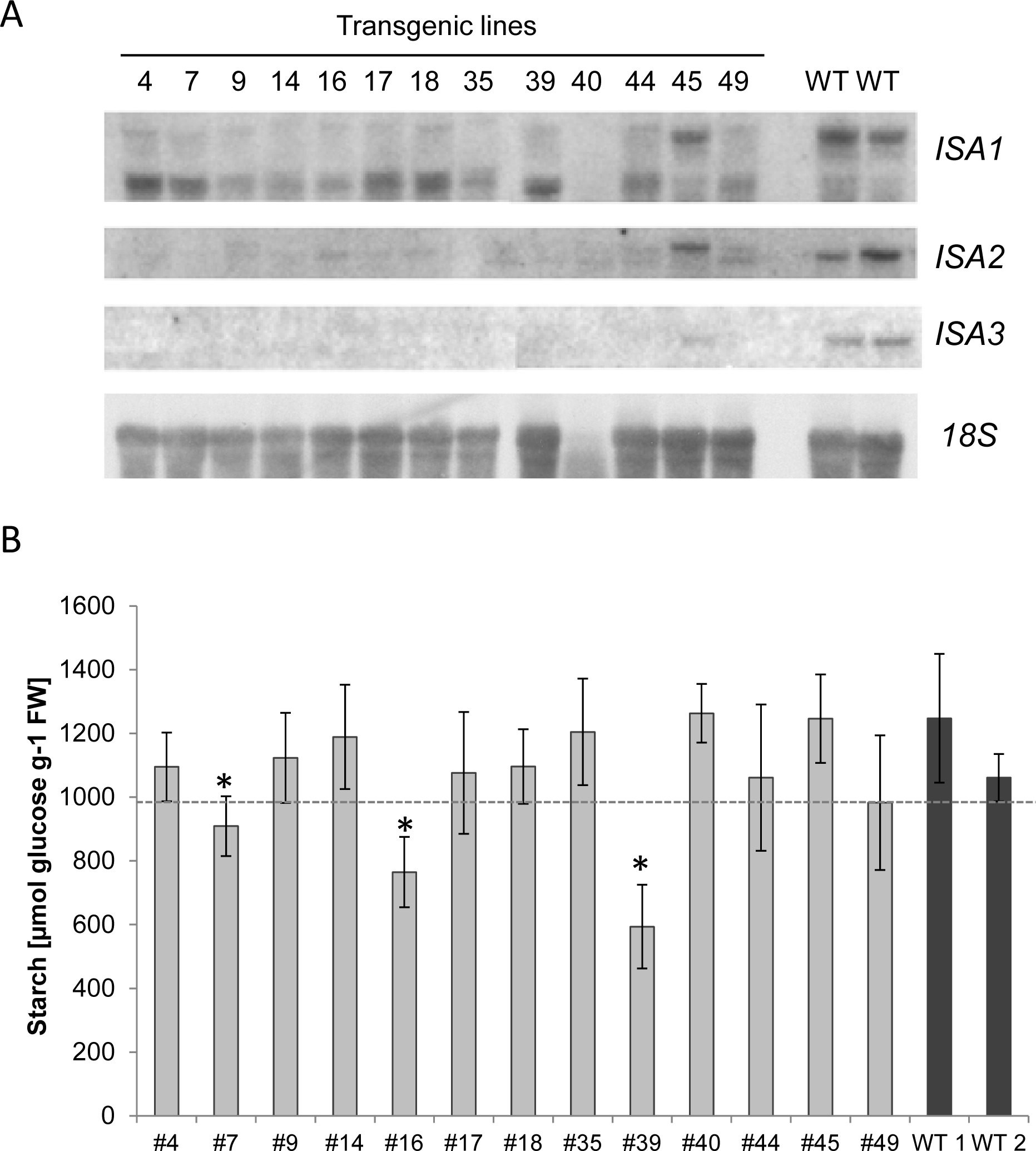

Supplement: S1 Fig — A) Analysis of transgenic lines by northern blotting. Total RNA was extracted from 13 pre-selecetd transgenic lines. Twenty μg of total RNA was separated in a formaldehyde-containing agarose gel and blotted onto nitrocellulose membrane. The membrane was consecutively probed with ISA1, ISA2 and ISA3 specific [32]P-labelled probes and a with 18S rRNA probe as loading control. B) Tuber starch content in transgenic lines. Two replicates were taken from 5 tubers per line after harvest. Values are the mean +/- SE. Asterisks indicate statistically significant differences at 5% level (* p < 0.05). (TIF) [file pone.0181444.s001.tif]

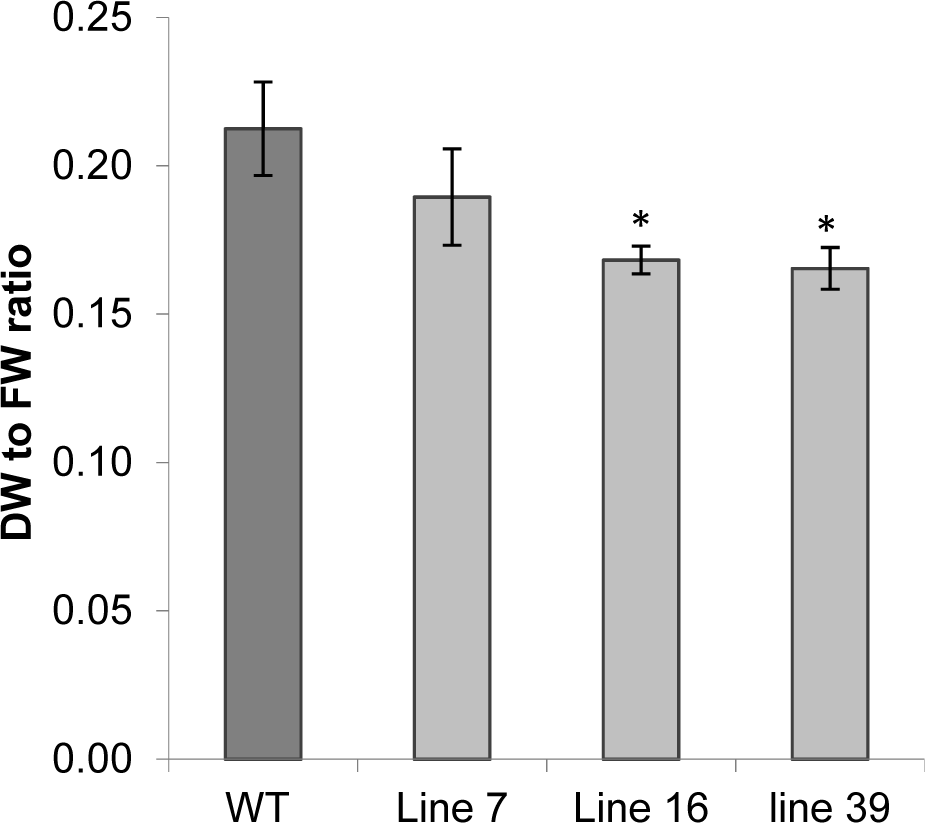

Supplement: S2 Fig — Slices were taken from 5 tubers per line and the fresh weight was determined. Subsequently, slices were dried in an oven at ca. 60°C for 2 days and weighed. Values are the mean +/- SE. Asterisks indicate statistically significant differences at 5% level (* p < 0.05). (TIF) [file pone.0181444.s002.tif]
